# Effect of the nitrification inhibitor 3,4-dimethylpyrazole phosphate (DMPP) on N-turnover, the N_2_O reductase-gene *nosZ* and N_2_O:N_2_ partitioning from agricultural soils

**DOI:** 10.1038/s41598-020-59249-z

**Published:** 2020-02-12

**Authors:** Johannes Friedl, Clemens Scheer, David W. Rowlings, Evi Deltedesco, Markus Gorfer, Daniele De Rosa, Peter R. Grace, Christoph Müller, Katharina M. Keiblinger

**Affiliations:** 1 Institute for Future Environments, Queensland University of Technology, Brisbane, QLD 4000 Australia; 2 Institute for Meteorology and Climate Research, Atmospheric Environmental Research, Karlsruhe Institute of Technology (KIT), Garmisch-Partenkirchen, Germany; 3 University of Natural Resources and Life Sciences Vienna, Department of Forest and Soil Sciences, Institute of Soil Research, Vienna, Austria; 4 AIT Austrian Institute of Technology, Center for Health & Bioresources, Tulln, Austria; 5 Department of Plant Ecology (IFZ), Justus-Liebig University, Giessen, Germany; 6 School of Biology and Environmental Science, University College Dublin, Belfield, Dublin, Ireland

**Keywords:** Element cycles, Climate-change mitigation

## Abstract

Nitrification inhibitors (NIs) have been shown to reduce emissions of the greenhouse gas nitrous oxide (N_2_O) from agricultural soils. However, their N_2_O reduction efficacy varies widely across different agro-ecosystems, and underlying mechanisms remain poorly understood. To investigate effects of the NI 3,4-dimethylpyrazole-phosphate (DMPP) on N-turnover from a pasture and a horticultural soil, we combined the quantification of N_2_ and N_2_O emissions with ^15^N tracing analysis and the quantification of the N_2_O-reductase gene (*nosZ*) in a soil microcosm study. Nitrogen fertilization suppressed *nosZ* abundance in both soils, showing that high nitrate availability and the preferential reduction of nitrate over N_2_O is responsible for large pulses of N_2_O after the fertilization of agricultural soils. DMPP attenuated this effect only in the horticultural soil, reducing nitrification while increasing *nosZ* abundance. DMPP reduced N_2_O emissions from the horticultural soil by >50% but did not affect overall N_2_ + N_2_O losses, demonstrating the shift in the N_2_O:N_2_ ratio towards N_2_ as a key mechanism of N_2_O mitigation by NIs. Under non-limiting NO_3_^−^ availability, the efficacy of NIs to mitigate N_2_O emissions therefore depends on their ability to reduce the suppression of the N_2_O reductase by high NO_3_^−^ concentrations in the soil, enabling complete denitrification to N_2_.

## Introduction

Agricultural soils have become the main source of anthropogenic nitrous oxide (N_2_O), a powerful greenhouse gas and the single most important substance depleting stratospheric ozone^1^. Delaying the conversion of ammonium (NH_4_^+^) to nitrate (NO_3_^−^), nitrification inhibitors (NIs) have been suggested as a means to reduce N_2_O emissions from agricultural soils. NIs demonstrated their efficacy across different cropping soils^2^, but results vary widely, and in particular in pasture soils the use of NIs had no or little effect on N_2_O emissions^3–5^. Despite a growing body of research on NIs, mechanisms and factors determining their efficacy to reduce N_2_O emission remain poorly understood^6^. The challenges to understand these mechanisms derive from the fact that N_2_O is formed via several different pathways in the soil matrix^7^, tightly coupled to different processes of N supply and consumption^8^. Critically, N_2_O can be further reduced to N_2_ via the microbial-mediated process of denitrification, and the sole quantification of N_2_O as affected by NIs provides therefore only a limited insight into mechanisms of N_2_O mitigation using NIs.

Microbial metabolic pathways can contribute via a wealth of different processes to N_2_O production and consumption, i.e. the reduction to N_2_ in soils. Apart from abiotic processes, N_2_O formation can be categorized into nitrification-mediated pathways, denitrification and biotic formation of hybrid N_2_O^9^. Denitrification is generally assumed to be the main process contributing to overall N_2_O production from agricultural soils^7,10–12^ and is also the main process reducing N_2_O into environmentally benign N_2_ via the N_2_O reductase, the enzyme encoded by the functional *nosZ* gene. The reduction of N_2_O to N_2_ does not reduce overall N losses but limits the environmental impact of denitrification losses from agricultural soils. A reduction of N_2_O emissions by NIs can be attributed to (a) reduced N_2_O production via nitrification mediated pathways, (b) reduced N_2_O production via denitrification (c) increased consumption of N_2_O via denitrification, i.e., a shift in the N_2_O:N_2_ ratio towards N_2_. As these effects may overlap, a mechanistic understanding of the effects of NIs on N_2_O production and consumption processes needs to be based on N_2_O source partitioning, and the direct quantification of N_2_.

Most of the NIs inhibit the first and rate-limiting enzymatic step of nitrification, the conversion of NH_4_^+^ to hydroxylamine (NH_2_OH) via the ammonia monooxygenase^13^. The inhibition of nitrification means a reduced supply of N into the NO_3_^−^ pool as the source pool of denitrification, but also an increase in NH_4_^+^ availability, leading to an increase of fertilizer N immobilization^11^ and mineralization/immobilization turnover rates^14,15^. Availability of N for N_2_O producing processes determines both production, but also consumption of N_2_O, as high NO_3_^−^ availability shifts the N_2_O:N_2_ ratio of denitrification towards N_2_O^16^. The link between N transformation rates and N_2_O and N_2_ emissions is therefore critical to understand the effects of NIs in agricultural soils.

Typically, pulses of N_2_O are observed after fertilization and irrigation events. These pulses are short-lived and can account for more than 90% of cumulative N_2_O emissions from agro-ecosystems^17^, defining the critical time-window which determines the efficacy of NIs to mitigate N_2_O emission. Building on extensive research at the field scale conducted across different agro-ecosystems^5,11,18–20^, this study investigated the short-term effect of 3,4-dimethylpyrazole phosphate (DMPP) on N-turnover and N_2_O and N_2_ emissions from two contrasting agricultural soils in response to N-fertilization. We combined a ^15^N tracing analysis with the direct quantification of N_2_ and N_2_O emissions using the ^15^N gas flux method, complemented with the quantification of the *nosZ* gene via quantitative polymerase chain reaction (qPCR) in a soil microcosm study to constrain factors determining the efficacy of the NI DMPP to mitigate N_2_O emissions from agricultural soils.

## Results

Physical and chemical properties for the two soils used in this experiment are shown in Table 1. The contrasting soils, a horticultural and a pasture soil, are henceforth referred to as sandy clay loam (sandy CL) and loam, according to their texture from 0–10 cm.

**Table 1:** Selected soil characteristics (0–10 cm) for a horticultural (Sandy clay loam) and a pasture soil (Loam) from subtropical Australia.

Soil property	Sandy clay loam- Horticulture soil	Loam - Pasture soil
Texture (USDA) (0–10 cm)	Sandy clay loam	Loam
Site	Gatton	Gympie
Latitude	−27.54	−26.19
Longitude	152.32	152.74
Mean annual rainfall	773 mm	1127 mm
Soil type (ASC)	Dermosol	Dermosol
Soil type (FAO)	Udic Argiustoll	Ferric Acrisol
Sand (%)	50.5	47.2
Silt (%)	22.8	38.8
Clay (%)	30.7	20.4
pH	7.4	6.1
Organic Carbon (%)	1.0	4.9
Total Nitrogen (%)	0.08	0.5
C:N ratio	12.5	9.8

### Nitrogen transformations and soil microbial parameters

**Figure 1:**
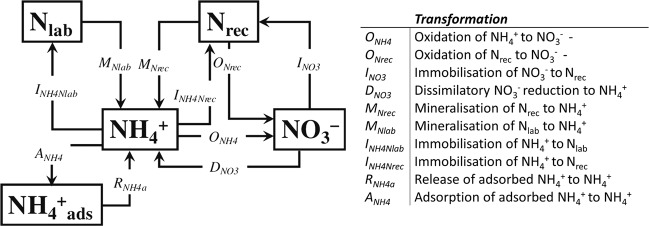
Conceptual ^15^N tracing model for the analysis of N gross transformations with the respective N transformations.

Gross N transformation rates were quantified with a ^15^N tracing model (Fig. 1) and differed markedly between soils when N-fertilizer was applied without the NI DMPP, referred to as the fertilizer only treatment (Table 2). Gross mineralization rates (*M*_*tot*_) in the loam exceeded those in the sandy CL by a factor of 39. In the loam, *M*_*tot*_ was dominated by the mineralization of labile N (*M*_*Nlab*_), while the mineralization of recalcitrant organic N (*M*_*rec*_) dominated in the sandy CL. Gross nitrification (*Nit*_*tot*_) was higher in the loam with 18.7 ± 0.03 μg N g^−1^ soil day^−1^ compared to 5.8 ± 0.03 μg N g^−1^ soil day^−1^ in the sandy CL. Autotrophic nitrification (*O*_*NH4*_) was the main pathway of NO_3_^−^ production in both soils, as heterotrophic nitrification of organic N (*O*_*Nrec*_) accounted for only 7% of *Nit*_*tot*_ in the sandy CL, and was negligible for *Nit*_*tot*_ in the loam. Immobilization of NH_4_^+^ (*I*_*NH4tot*_) and NO_3_^−^ (*I*_*NO3*_) was higher in the sandy CL compared to the loam, and was dominated by *I*_*NO3*_. In the sandy CL, only minor amounts of NO_3_^−^ were recycled in the NH_4_^+^ pool via dissimilatory NO_3_^−^ reduction to NH_4_^+^ (DNRA, referred to as *D*_*NO3*_ in the ^15^N tracing model), while *D*_*NO3*_ contributed with more than 2 μg N g^−1^ soil day^−1^ to NH_4_^+^ production in the loam. Microbial C (C_mic_) and N (N_mic_) as indicators for the size of the soil microbial biomass (SMB) were higher in the loam, exceeding C_mic_ and N_mic_ in the sandy CL by a factor of 5 and 7, respectively (Table 3).

**Table 2:** Gross soil N transformations (average ± standard deviation) in a horticultural (Sandy clay loam) and a pasture soil (Loam) after the application of NH_4_NO_3_ with and without the nitrification inhibitor DMPP. Means denoted by a different letter indicate significant differences for a specific N transformation across soils and treatments (i.e. no overlap of 95% confidence intervals).

N – transformation μg N g^−1^ soil day^−1^		Sandy clay loam - Horticulture soil					Loam -Pasture soil				
		−DMPP		+DMPP		**DMPP effect**	−DMPP		+DMPP		**DMPP effect**
			↔		↔			↔		↔	
Mineralisation of *N*_*rec*_ to NH_4_^+^	*M*_*Nrec*_	0.12 ± 0.04	c	1.03 ± 0.11*	b	+ 776%	2.59 ± 0.09	a	2.50 ± 0.11	a	
Immobilisation of NH_4_^+^ to *N*_*rec*_	*I*_*NH4-Nrec*_	0.16 ± 0.04	b	0.79 ± 0.22*	a	+ 397%	0.002 ± 0.0005	c	0.0020 ± 0.00005	c	
Mineralisation of *N*_*lab*_ to NH_4_^+^	*M*_*Nlab*_	0.10 ± 0.03	d	0.25 ± 0.05*	c	+ 166%	5.80 ± 0.28*	a	5.37 ± 0.07	b	−7%
Immobilisation of NH_4_^+^ to *N*_*lab*_	*I*_*NH4-Nlab*_	0.81 ± 0.26	b	2.59 ± 0.50*	a	+ 220%	0.002 ± 0.0002	c	0.002 ± 0.0002	c	
Oxidation of *N*_*rec*_ to NO_3_^−^	*O*_*Nrec*_	0.38 ± 0.11	a	0.34 ± 0.22	a		0		0		
Immobilisation of NO_3_^−^ to *N*_*rec*_	*I*_*NO3*_	9.48 ± 0.12	a	6.55 ± 0.31*	b	−31%	0.017 ± 0.0005	c	0.016 ± 0.0005	c	
Oxidation of NH_4_^+^ to NO_3_^−^	*O*_*NH4*_	5.44 + 0.28	c	2.04 ± 0.20*	d	−63%	18.64 ± 0.24*	a	17.46 ± 0.37	b	−6%
Dissimilatory NO_3_^−^ reduction to NH_4_^+^	*D*_*NO3*_	0.026 ± 0.003	d	0.14 ± 0.01*	c	+ 431%	2.14 ± 0.05	a	2.02 ± 0.08	a	
Adsorption of adsorbed NH_4_^+^ to NH_4_^+^_ads_	*A*_*NH4*_	1.18 ± 0.22	a	0.87 ± 0.75	a		0		0		
Release of adsorbed NH_4_^+^ to NH_4_^+^	*R*_*NH4a*_	0.08 ± 0.02	b	0.68 ± 0.07*	a	+ 714%	0		0		
Total mineralisation *M*_*nrec*_ + *M*_*nlab*_	*M*_*tot*_	0.21 ± 0.05	d	1.29 ± 0.12*	c	+ 502%	8.39 ± 0.29	a	7.87 ± 0.13*	b	−6%
Total nitrification *O*_*Nrec* + _*O*_*NH4*_	*Nit*_*tot*_	5.82 ± 0.30	c	2.39 ± 0.30*	d	−59%	18.64 ± 0.24	a	17.46 ± 0.37*	b	−6%
Total NH_4_^+^ immobilisation	*I*_*NH4tot*_	0.97 ± 0.31	b	3.37 ± 0.72*	a	+ 249%	0.004 ± 0.001	c	0.004 ± 0.0001	c	
Contribution of *M*_*nlab*_ to *M*_*tot*_	*M*_*nlab*_*/M*_*ntot*_	45%		20%			69%		68%		
Contribution of *O*_*NH4*_ to *Nit*_*tot*_	*O*_*NH4*_*/ Nit*_*tot*_	93%		86%			100%		100%		

*denotes a significant effect of DMPP

Letters denote significant differences for a specific N transformation across soils and treatments (i.e. no overlap of 95% confidence intervals).

**Table 3:** Soil mineral N concentrations 30 minutes and 48 hours after N fertilizer application with and without the nitrification inhibitor DMPP; and dissolved organic C and soil microbial C and N prior and 48 hours after fertilizer application with and without DMPP in a horticulture and a pasture soil.

		time		Sandy clay loam Horticulture soil		Loam Pasture soil		
					↓		↓	↔
NH_4_^+^	μg N g^−1^ soil	30 min	after fertilization	17.0 ± 0.1	a	18.2 ± 0.2	a	*
		48 h	Fertilizer	9.9 ± 0.5	c	2.1 ± 0.1	c	*
		48 h	Fertilizer + DMPP	14.4 ± 0.2	b	7.1 ± 0.8	b	*
			**DMPP effect**	**+42%**		**+223%**		
NO_3_^−^	μg N g^−1^ soil	30 min	after fertilization	70.9 ± 2.2	a	135.2 ± 1.4	b	*
		48 h	Fertilizer	70.2 ± 2.1	a	175.0 ± 3.3	a	*
		48 h	Fertilizer + DMPP	61.9 ± 1.4	b	171.0 ± 4.0	a	*
			**DMPP effect**	**−12%**		—		
DOC	μg C g^−1^ soil	0	prior fertilization	37.7 ± 1.3	c	146.1 ± 2.0	c	*
		48 h	Fertilizer	71.3 ± 4.1	b	197.9 ± 9.4	b	*
		48 h	Fertilizer + DMPP	107.3 ± 12.0	a	261.3 ± 6.5	a	*
			**DMPP effect**	**+50%**		**+32%**		
Microbial C	μg C_mic_ g^−1^ soil	0	prior fertilization	93.6 ± 18.7	a a a	433.0 ± 34.4	b	*
		48 h	Fertilizer	61.5 ± 13.9	b b	471.8 ± 13.5	a	*
		48 h	Fertilizer + DMPP	66.3 ± 7.4	b	480.3 ± 7.5	a	*
			**DMPP effect**	—		—		
Microbial N	μg N_mic_ g^−1^ soil	0	prior fertilization	11.9 ± 0.6	a a	89.1 ± 16.8	a	*
		48 h	Fertilizer	13.9 ± 1.9	a	82.3 ± 2.5	a	*
		48 h	Fertilizer + DMPP	11.9 ± 0.8	a	92.7 + 4.3	a	*
			**DMPP effect**	—		—		

Letters denote significant differences between treatments within a soil.

*denote significant differences (P < 0.05) between soils within a treatment.

### Effect of DMPP on N-transformations and soil parameters

The application of N-fertilizer with DMPP had no significant effect on N transformations in the loam but changed N-turnover dynamics in the sandy CL (Table 2). DMPP reduced *O*_*NH4*_ only by 6% in the loam, but reduced *O*_*NH4*_ by more than 60% in the sandy CL. In the sandy CL, both *M*_*tot*_ and *I*_*NH4tot*_ increased, as well as the relative contribution of *M*_*Nrec*_ to *M*_*tot*_, accounting for 80% of *M*_*tot*_. *I*_*NO3*_ decreased by 31%, while *D*_*NO3*_ increased by a factor of >5. DMPP did not affect the soil microbial biomass (SMB) but increased dissolved organic carbon (DOC) by 50% and 32% in the sandy CL and loam, respectively (Table 3).

### Emissions of N_2_O and N_2_

**Figure 2:**
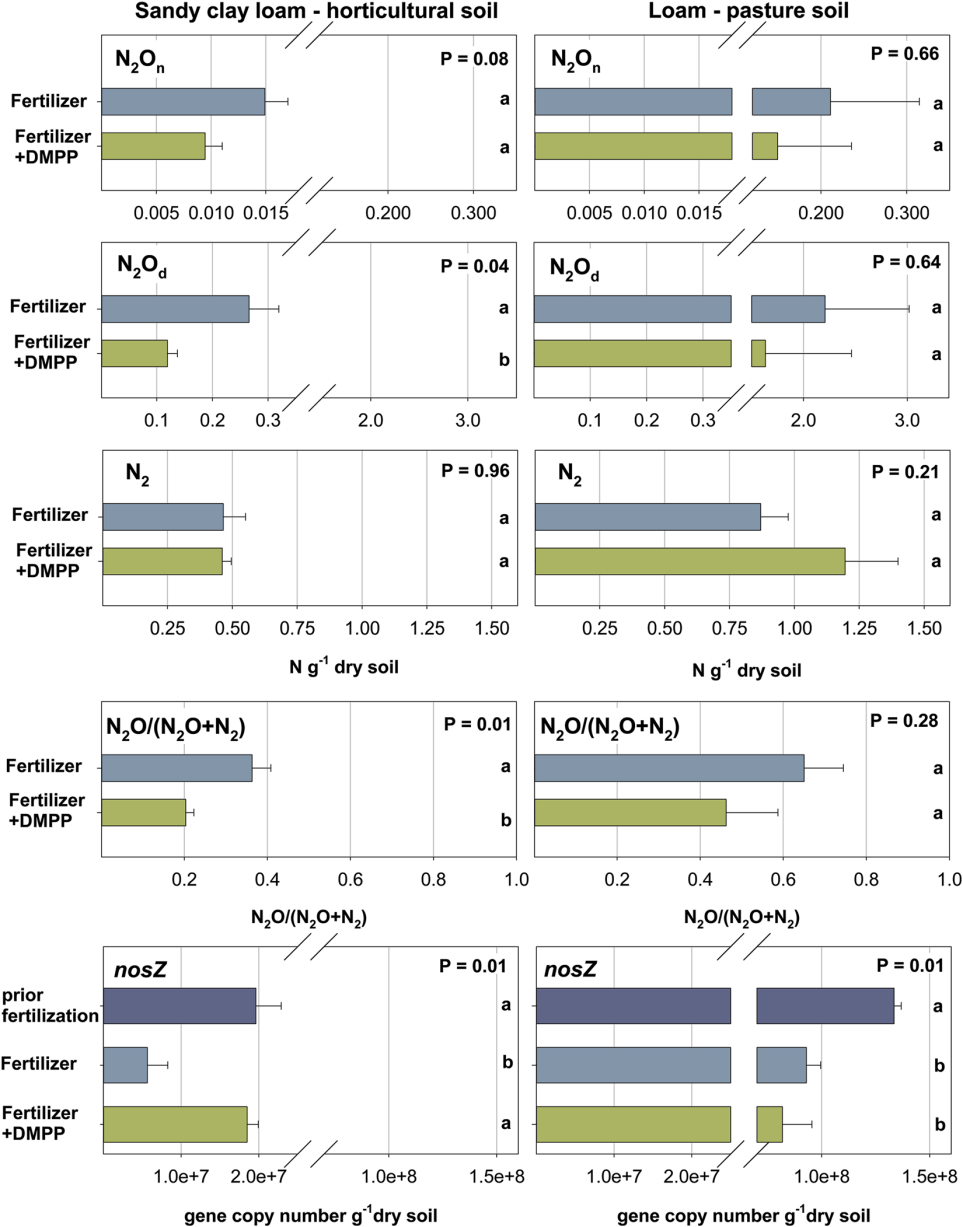
Cumulative emissions of N_2_O derived from nitrification (N_2_O_n_) and denitrification (N_2_O_d_), cumulative N_2_ emissions, the product ratio of denitrification (N_2_O/(N_2_O_d_ + N_2_) and the abundance of the *nosZ* gene encoding the N_2_O reductase from a horticultural soil (Sandy clay loam) and a pasture soil (Loam) after the application of NH_4_NO_3_ with and without the nitrification inhibitor DMPP.

The dominant N_2_O production pathway in both soils was denitrification, accounting for more than 90% of the N_2_O produced (Fig. 2). Over 48 hours, 0.24 ± 0.03 and 1.46 ± 0.38 μg N_2_O - N g^−1^ soil were emitted from the sandy CL and the loam, respectively. Both N_2_O emissions via denitrification (N_2_O_d_) and nitrification (N_2_O_n_) were higher from the loam, exceeding those from the sandy CL by a factor of >8 (Fig. 2). Over the two day incubation period, 0.47 ± 0.09 μg N_2_ - N g^−1^ soil and 0.87 ± 0.11 μg N_2_ - N g^−1^ soil were emitted as N_2_ from the sandy CL and the loam, respectively. The main product of denitrification (N_2_O_d_ + N_2_) from the sandy CL was N_2_, with N_2_O_d_ accounting for 36% of total denitrification losses. Denitrification losses from the loam however were dominated by N_2_O_d_, accounting for 75% of total denitrification. There was no indication for hybrid production of N_2_O or N_2_.

### The response of the N_2_O reductase gene *nosZ* to fertilization and the use of DMPP

The abundance of *nosZ* prior to fertilization differed markedly between soils (Fig. 2). Copy numbers of *nosZ* in the loam exceeded those in the sandy CL by a factor of 6. After fertilization and the increase in soil moisture from 50% to 75% water-filled pore space (WFPS), *nosZ* copy numbers decreased in both soils, with a reduction by 77% and 32% for the sandy CL and the loam, respectively. DMPP did not affect *nosZ* abundance in the loam. DMPP however increased *nosZ* copy numbers by 227% compared to the fertilizer only treatment in the sandy CL.

### Effect of DMPP on N_2_O and N_2_ emissions

DMPP significantly reduced N_2_O emission from the sandy CL but had no effect on N_2_O emissions from the loam (Table 4). DMPP reduced N_2_O_d_ from the sandy CL by 46% (P < 0.05), but did not affect N_2_O_n_ (Fig. 2). There was no effect of DMPP on N_2_ emissions from the two soils. In the sandy CL, DMPP shifted the product ratio of denitrification (N_2_O_d_ /(N_2_O_d_ + N_2_)) to N_2_, decreasing the percentage of denitrification emitted as N_2_O_d_ from 36% to 20%.

**Table 4:** Cumulative emissions of N_2_, N_2_O and CO_2_ from a horticultural soil (Sandy clay loam) and a pasture soil (Loam) after the application of NH_4_NO_3_ with and without the nitrification inhibitor DMPP.

		Fertilizer			Fertilizer + DMPP			DMPP effect	
		Sandy clay loam	Loam		Sandy clay loam	Loam		Sandy clay loam	Loam
		Horticulture soil	Pasture soil		Horticulture soil	Pasture soil		Horticulture soil	Pasture soil
Denitrification	μg N_2_ + N_2_O_d_ - N g^−1^ soil	0.73 ± 0.13	3.08 ± 0.87	P = 0.04	0.58 ± 0.05	2.83 ± 1.02	P = 0.07	P = 0.32	P = 0.86
N_2_ emissions	μg N_2_ - N g^−1^ soil	0.47 ± 0.09	0.87 ± 0.11	P = 0.03	0.46 ± 0.04	1.20 ± 0.20	P = 0.04	P = 0.96	P = 0.21
N_2_O emissions	μg N_2_O - N g^−1^ soil	0.24 ± 0.03	1.46 ± 0.38	P = 0.01	0.14 ± 0.02	1.80 ± 0.52	P = 0.01	**−54%** /P = 0.01	P = 0.60
CO_2_ emissions	μg CO_2_ - C g^−1^ soil	6.55 ± 0.52	44.66 ± 1.73	P = 0.01	5.99 ± 0.18	46.27 ± 1.35	P < 0.01	P = 0.35	P = 0.47

## Discussion

The fertilization and irrigation of agricultural soils triggers a cascade of N transformations associated with pulses of N_2_O and N_2_ emissions. These short-term events are critical to understand the effects of NIs on N_2_O production and consumption in agricultural soils. Linking N turnover to emissions of N_2_O and N_2_ and the abundance of the N_2_O reductase gene *nosZ* in a short-term incubation demonstrated (a) that increasing NO_3_^−^ availability after fertilization suppressed *nosZ* abundance, (b) that *nosZ* abundance, nitrification and N_2_ + N_2_O emissions remained largely unaffected by DMPP in the loam and (c) that DMPP decreased nitrification and increased *nosZ* abundance in the sandy CL, shifting the N_2_:N_2_O ratio towards N_2_. Our findings highlight the short-term effect of DMPP as highly soil specific, and show that reduced nitrification by DMPP can limit the suppression of the N_2_O reductase by high NO_3_^−^ concentrations in the soil, enabling complete denitrification to N_2_.

Nitrogen transformation rates identified the loam as the more active soil regarding N turnover compared to the sandy CL (Table 2). Gross mineralization rates (*M*_*tot*_) of more than 8 μg N g^−1^ soil day^−1^ together with a low immobilization of mineral N (*I*_*NH4tot*_ and *I*_*NO3*_) denote high mineral N availability due to the rapid mineralization of organic N. This is further supported by the dominant contribution of the labile organic N pool to mineralization (*M*_*Nlab*_), representing the microbial biomass and low molecular organic N compounds with a fast turnover. The high nitrification rates in the loam (>18 μg N g^−1^ soil day^−1^) denote rapid conversion of mineralized N to NO_3_^−^ and show the dominant role of NH_4_^+^ oxidation for N-turnover in this soil. Gross mineralization was markedly lower in the sandy CL with *M*_*tot*_ at only 0.21 μg N g^−1^ soil day^−1^ and dominated by the mineralization of recalcitrant organic N, indicating limited and slower supply of mineral N via mineralization. Mineralization accounted for only 4% of nitrified N in the sandy CL, as compared to 45% in the loam, implying a rapid depletion of the NH_4_^+^ pool in the sandy CL. The observed differences between soils are consistent with microbial C and N contents (Table 3), indicating a larger soil microbial biomass in the loam and reflect the impact of perennial versus short term/annual and tilled versus undisturbed plant-systems on soil organic matter and microbial activity: Intensive tillage and irrigation in horticultural systems lead to loss of soil organic C^21^, while an extensive root system under permanent pasture is likely to promote microbial activity through constant inputs of C and N. These findings establish the differences in magnitude and relative importance of N transformations and microbial activity between the two contrasting soils.

The main source of N_2_O in both soils was denitrification, accounting for more than 90% of N_2_O produced (Fig. 2), which is in line with previous results from both field^11^ and laboratory studies^10,12^. The ability of soils to act as an N_2_O sink, i.e. the trait to reduce N_2_O to N_2_ has been linked to the abundance of *nosZ*, used as proxy for microorganisms involved in the reduction of N_2_O. In the study presented here, we compared *nosZ* abundance with direct measurements of N_2_ and N_2_O, evaluating the influence of DMPP on of microorganisms reducing N_2_O. The abundance of *nosZ* prior to fertilizer addition was higher in the loam, which is consistent with the reported positive correlation of *nosZ* copy numbers with soil organic C^22^. The synthesis of the N_2_O reductase is promoted by anoxic conditions^23^, and the increase in soil moisture together with the addition of fertilizer should have increased *nosZ* abundance. However, *nosZ* abundance decreased in both soils in the fertilizer only treatment (Fig. 2), indicating that increased NO_3_^−^ availability due to fertilization and nitrification promoted the reduction of NO_3_^−^ rather than N_2_O, shifting the N_2_O_d_/(N_2_O_d_ + N_2_) ratio towards N_2_O. The magnitude and N_2_O:N_2_ partitioning of denitrification losses is consistent with the nitrification rates in both soils and as such shows the N_2_O_d_/(N_2_O_d_ + N_2_) ratio as a function of soil intrinsic N – turnover. Cumulative N_2_O_d_ losses of >2 μg N g^−1^ soil and 75% of denitrification (N_2_O_d_ + N_2_) emitted as N_2_O from the loam show increased substrate availability for denitrification and simultaneous suppression of *nosZ* abundance by high NO_3_^−^ availability (Fig. 2). In turn, lower denitrification losses with only 36% emitted as N_2_O_d_ reflect slower N turnover in the sandy CL. These findings suggest that the suppression of the N_2_O reductase and increased N substrate availability are responsible for the large pulses of N_2_O from agricultural soils observed after fertilization and irrigation. Our results denote an increased risk of N_2_O loss from highly productive agricultural soils^19^, where increased mineralization of soil organic N due to fertilization, i.e., priming is likely to amplify the preferential reduction of NO_3_^−^, and as such the production of N_2_O via denitrification.

DMPP reduced N_2_O emissions from the sandy CL by more than 54% (Table 4). This is reflected in DMPP’s effect on autotrophic nitrification (*O*_*NH4*_) showing a reduction of 63% in the sandy CL (Table 2). The minor reduction of *O*_*NH4*_ by DMPP had however no effect on N_2_O emissions from the loam. In both soils, N_2_O derived from nitrification mediated pathways accounted for less than 15% of overall N_2_O, showing no response to the DMPP treatment. For the sandy CL, this suggests that DMPP primarily affected N_2_O production pathways indirectly, that is by reducing NO_3_^−^ availability for denitrification, demonstrated by the reduction of N_2_O derived from denitrification by 46%. DMPP increased *nosZ* abundance in the sandy CL by a factor >2 compared to the fertilizer only treatment (Fig. 2). In the absence of direct N_2_ measurements, this effect has been interpreted as a shift of denitrification losses towards N_2_^24^. Experimental evidence linking increased *nosZ* abundance with DMPP to N_2_ and N_2_O emissions^25^ is based on the acetylene inhibition method, which has been shown to lead to an irreproducible underestimation of denitrification rates^9^. Furthermore, acetylene itself is a potent NI, questioning the use of this method when investigating the effects of NIs on the magnitude and the N_2_O_d_/(N_2_O_d_ + N_2_) ratio of denitrification. In the study presented here, DMPP reduced the N_2_O_d_/(N_2_O_d_ + N_2_) ratio by 44% in the sandy CL, demonstrating a significant shift towards N_2_ (Fig. 2). These results link the increase of *nosZ* abundance in response to DMPP in the sandy CL to a shift in the N_2_O_d_:N_2_ ratio towards N_2_, based on direct measurements of N_2_ and N_2_O_d_ using the ^15^N gas flux method. In contrast to previous incubation studies investigating N_2_O:N_2_ partitioning in response to DMPP^26,27^, emissions of N_2_O and N_2_ were quantified after incubation under atmospheric O_2_ conditions and without adding an easily available C source to stimulate denitrification, as these conditions would have altered short-term N dynamics in response to DMPP. Importantly, the shift towards N_2_ was not observed for the loam, where DMPP had a negligible effect on nitrification. Our findings indicate that the reduction of nitrification by DMPP in the sandy CL reduced the suppression of the N_2_O reductase after fertilization, enabling complete denitrification to N_2_. Emissions of N_2_O produced via nitrification mediated pathways were not affected by DMPP in this soil, showing the reduction of N_2_O emissions by DMPP as an indirect effect limiting NO_3_^−^ availability for denitrification.

The spatial coverage of nitrifying microsites by the inhibitor is critical for efficient inhibition of nitrification. Limited diffusion of DMPP may explain the he observed inefficacy of DMPP to reduce autotrophic nitrification in the loam, which is consistent with reports from other pasture soils^15^. The amount of DMPP applied with N fertilizer is small, and the initial sorption to organic matter and uneven distribution of DMPP may hinder its short-term effectiveness to reduce nitrification in specific micro sites. Sorption of DMPP is likely to be more pronounced in the loam as a pasture soil with higher organic matter content as compared to the sandy CL owing to the positive correlation of DMPP sorption with organic C^28,29^. The high microbial activity in the loam also infers a larger number of microsites with nitrifying activity compared to the sandy CL, suggesting the spatial separation of DMPP from nitrifiers may be responsible for the short-term inefficacy of DMPP to reduce autotrophic nitrification in the loam. This theory is further supported by a study where DMPP did not affect the initial pulse of N_2_O after fertilization and irrigation from the loam, but reduced denitrification losses after that initial period^11^. This shows a delayed effect of DMPP in this soil, demanding further research on how diffusion in the soil matrix, sorption and distribution affects DMPPs efficacy to reduce autotrophic nitrification.

DMPP also affected non-targeted N transformation in the sandy CL: Mineralization and immobilization turnover was stimulated by DMPP, demonstrated by the five-fold increase of total mineralization (*M*_*nrec*_ + *M*_*nlab*_) and the simultaneous increase of NH_4_^+^ immobilization (*I*_*NH4rec*_ + *I*_*NH4lab*_) by a factor > 2 (Table 2). Increased mineralization/immobilization turnover has been reported after the application of DMPP^15^ and dicyandiamide (DCD)^14^ and can be attributed to higher NH_4_^+^ availability, stimulating microbial immobilization of NH_4_ (*I*_*NH4lab*_) and mineralization of labile N_org_ (*M*_*Nlab*_) to NH_4_^+^. This effect may further prime the mineralization of recalcitrant N (*M*_*Nrec*_) in response to DMPP^30^. Interestingly, DMPP increased DOC availability in both soils, confirming previous results from a wheat-maize cropping system^31^ (Table 3). Increased *M*_*Nrec*_ in the sandy CL indicates mineralization of organic matter induced by DMPP contributed to higher DOC availability, but no such effect was observed for *M*_*Nrec*_ in the loam. Based on the data available, it remains unclear what caused the increase in DOC in response to DMPP. This increase has however important implications for N-turnover, in particular for the sandy CL as soil with limited labile C availability. DMPP increased DNRA by a factor >5 in the sandy CL, suggesting labile C promoted NO_3_^−^ consumption via DNRA^10,23^. DNRA competes with denitrification for available NO_3_^−^, but the magnitude of DNRA in the sandy CL was insignificant regarding NO_3_^−^ availability for denitrification. More importantly, labile C affects denitrification^32^, by supplying a reductant for denitrifiers, or through the stimulation of heterotrophic soil respiration, decreasing soil O_2_ levels and thus promoting denitrification. Furthermore, readily decomposable C can decrease the N_2_O_d_/(N_2_O_d_ + N_2_) ratio of denitrification^23^. The increase in DOC observed in this study demonstrates an important non-targeted effect of DMPP, which can alter both rate and N_2_O:N_2_ partitioning of denitrification losses and therefore warrants further research.

Nitrification activity during pre-incubation increased NO_3_^−^ levels in both soils. In the loam, NO_3_^−^ levels were above those measured at the respective field site, which is also reflected in higher N_2_O_d_/(N_2_O_d_ + N_2_) ratios^11^. This phenomenon often occurs in incubation studies, where the absence of plant uptake, pre-incubation^33,34^, and the addition of glucose^26^ increases NO_3_^−^ levels in the soil. It is therefore important to consider N substrate availability when interpreting the effects of NIs on rate and N_2_O:N_2_ partitioning of denitrification losses. The mineral N levels in both soils indicate no N substrate limitation for denitrification regardless of the treatment. Under these conditions, DMPP had no effect on overall denitrification losses in both soils. The minor reduction of nitrification by DMPP in the loam did not reduce NO_3_^−^ availability to a degree that limited preferential reduction of NO_3_^−^. The high initial NO_3_^−^ values in the loam are also likely to have overwritten a significant reduction of nitrification. The reduction of N_2_O emissions, together with the increase of *nosZ* abundance in the sandy CL suggests however that DMPP lowered NO_3_− availability below a soil specific treshold^35^, limiting the preferential reduction of NO_3_^−^ over N_2_O. The results from the sandy CL confirm the proposed mechanism of N_2_O reduction via a shift in the N_2_:N_2_O ratio^26^, and show that DMPPs inhibitory effect on nitrification can limit the suppression of the N_2_O reductase, promoting complete denitrification to N_2_.

The demonstrated link between *nosZ* and directly measured N_2_O and N_2_ emissions suggests that DMPP promotes the abundance of *nosZ* carrying denitrifiers. Including a comprehensive assessment of abundance and activity of nitrifying and denitrifying microbial communities in future research could further help to understand mechanisms of N_2_O mitigation by DMPP. Our study shows N dynamics in response to DMPP on a soil microcosm scale. This approach does not account for plant-microbe interactions and plant N uptake under field conditions but enables to isolate effects of NIs on key N transformations, with practical implications for the use of NIs in different agricultural soils. The relative magnitude of N_2_O emissions reflects cumulative losses observed from the same soils in the field, demonstrating a larger N_2_O mitigation potential for the pasture soil as compared to the horticultural soil. The short term inefficacy of DMPP to reduce nitrification in the pasture soils demands therefore improved strategies regarding rate and application of NIs. In soils with high organic matter content, and high soil intrinsic N turnover, repeated applications of DMPP, increasing the rate of DMPP, and/or the application of DMMP prior to fertilization may increase DMPPs efficacy, limiting the effect of N fertilizer priming on N_2_O emissions. Decreased *nosZ* abundance after fertilization and irrigation indicates suppression of the N_2_O reductase by increased NO_3_^−^ availability, identifying NO_3_^−^ availability as the control for the reduction of NO_3_^−^ vs. N_2_O, which determines the magnitude of N_2_O losses. These findings apply to conditions of non-limiting NO_3_^−^ availability for overall denitrification, which can be found in agricultural soils after N fertilization and irrigation when plant N uptake is limited. Under these conditions, the efficacy of NIs to mitigate N_2_O emissions depends on their ability to limit the suppression of the N_2_O reductase by high NO_3_^−^ concentrations in the soil, enabling complete denitrification to N_2_.

## Material and Methods

### Soils and site

Soil samples (0–10 cm) were collected randomly (n = 4) from a vegetable cropping site (Gatton, Qld)^20^ and an intensively managed dairy pasture (Gympie, Qld)^11^ in subtropical Australia, referred to according to their texture in the first 10 cm as sandy clay loam (sandy CL) and loam, respectively. Site characteristics including physical and chemical soil properties are shown in Table 1. Soil samples were bulked, air dried and sieved to <4 mm and stored in a cold room at 4 °C.

### Soil microcosms

Before treatment application, the soils were incubated in bulk for 4 days at a gravimetric water content of 30%. The experimental design consisted of two soils and two treatments: ammonium nitrate (NH_4_NO_3_) and NH_4_NO_3_ with DMPP (DMPP), each with four different ^15^N label combinations and four replicates. The NH_4_NO_3_ was applied with either (a) the NH_4_^+^ (^15^NH_4_NO_3_^−^) or (b) the NO_3_^−^ (NH_4_^15^NO_3_^−^) labeled at 10 atom %. NH_4_^15^NO_3_^−^ at 60 atom % (c) was used to quantify N_2_ emissions^36^, while non-labeled NH_4_NO_3_ (d) was used for the quantification of the SMB, DOC, and *nosZ* abundance. For the incubation, soil microcosms were established in centrifuge tubes (50 ml) using the equivalent of 8 g oven dry soil at a soil bulk density of 1 g cm^−3^. NH_4_NO_3_ equivalent to 35 µg N g^−1^ soil was applied in solution (1 ml) with 0.6% DMPP (w/w) added for the DMPP treatment. Additional water was applied to achieve the water-filled pore space (WFPS) of 75%. Water and fertilizer solutions were applied dropwise on two layers of 4 g of soil to ensure homogenous ^15^N labeling. After fertilization, centrifuge tubes were closed with Suba-seals (Sigma Aldrich) and were kept closed in an incubator at a constant temperature of 25 °C between gas sampling events. Additional soil microcosms (a and b, n = 4) were established for destructive sampling 30 minutes after fertilizer application.

### Soil analysis

#### Soil mineral N

All soil mineral N extractions were conducted in the centrifuge tubes to avoid subsampling errors using 40 ml 2 M KCl (1:5 w/v ratio). Four soil microcosms per soil were extracted before fertilizer application to determine initial conditions. Soil microcosms a and b were extracted with 40 ml 2 M KCl, 30 minutes (t = 0) and 48 h (t = 2 days) after N fertilizer application. The centrifuge tubes were shaken with a horizontal shaker (150 rpm) for one hour, and extracts were filtered through Whatman no. 42 filter paper. After sample dilution, concentrations of NH_4_^+^ and NO_3_^−^ were determined using colorimetric methods, NH_4_^+^ with a modified indophenol reaction^37^ and NO_3_^−^ with the VCL3/Griess assay^38^. The ^15^N enrichments of the NH_4_^+^ and NO_3_^−^ pool were determined for soil microcosms a and b by the diffusion method^39^.

#### Quantitative PCR analyses

For qPCR analysis, subsamples of 0.25 g of soil were taken prior to fertilizer application, and 48 h after (t = 2 days) from soil microcosms d and extracted immediately for total DNA using the PowerLyzer® PowerSoil® DNA Isolation Kit from MoBio (Mobio Laboratories, Inc., Carlsbad, CA, USA) according to the manufacturer’s instructions, with some minor modifications. Briefly, the soil was extracted twice by using the same soil and PowerBead Tubes to increase recovery of DNA. DNA concentration and quality were determined spectrophotometrically (NanoDrop 2000, Thermofisher, MA, USA). The two DNA aliquots from each sample were pooled before qPCR. The real-time PCR assay was carried out in a volume of 10 µl, and the assay mixture contained GoTaq® qPCR Master Mix (Promega, USA), 10 µM of each *nosZ* primer^40^ and 1 µl of pooled template DNA. Thermal cycling conditions for the nosZ2F (CGCRACGGCAASAAGGTSMSSGT) and *nos*Z2R (CAKRTGCAKSGCRTGGCAGAA) were as follows: an initial cycle of 95 °C for 3 min, 39 cycles of 95 °C for 15 s, 39 cycles of 60 °C for 45 s, 39 cycles of 72 °C for 45 s and 65 °C and 95 °C for 5 s. Each sample was quantified in triplicates using the iCycler iQ Real-Time PCR Detection System and the iQ 5 Optical System software (Bio-Rad Laboratories, Hercules, CA, USA).

#### Soil microbial biomass

Microbial C (C_mic_) and N (N_mic_) were quantified before and two days after fertilizer application using the chloroform fumigation-extraction^41^. Two aliquots of 3.5 g soil were sampled from each soil microcosm (d) with one aliquot subsequently fumigated with chloroform for 24 h. Fumigated and non-fumigated samples were extracted with 2 M KCl (1:10 w/v) and stored frozen until further analyses. Samples were acidified to remove inorganic C and analyzed for total N and organic C with an automated TOC/TN analyzer (TOC-V CPHE200V) linked with a TN-unit (TNM-1 220 V, Shimadzu Corporation, Kyoto, Japan). C_mic_ and N_mic_ were calculated as the difference in N and C between fumigated and non-fumigated samples without using a correction factor^42^. Dissolved organic C (DOC) was quantified as the amount of total C in the extracts of the non-fumigated samples.

### Gas sampling and analysis

Air samples (n = 4) were taken daily before closing the centrifuge tubes to quantify ambient N_2_O concentrations. Specific background samples were taken above the respective soil microcosms treated with NH_4_^15^NO_3_ at 60 atom % (c) for ^15^N_2_ analysis before closing the tubes. The entire headspace atmosphere was sampled 24 and 48 h after closure using a gas-tight syringe from soil microcosms a, b and c. After the 24 h gas sampling, the Suba-seals were removed for 10 minutes, allowing the headspace atmosphere to equilibrate^10^. Gas samples were transferred into pre-evacuated 12 ml exetainer tubes with a double wadded Teflon/silicon septa cap (Labco Ltd, Buckinghamshire, UK) and stored until N_2_O and CO_2_ analysis by gas chromatography (Shimadzu GC-2014). Gas samples from soil microcosms c were also analyzed for the isotopologues of N_2_ (^15^N^14^N, ^15^N^15^N) and N_2_O ([^14^N^15^N^16^O + ^15^N^14^N^16^O] and ^15^N^15^N^16^O) using an automated isotope ratio mass spectrometer (IRMS) coupled to a trace gas preparation unit (Sercon Limited, 20–20, UK).

### Fluxes of N_2_, N_2_O and CO_2_

The triple labelling approach generates gas samples from three ^15^N fertilizer treatments with four replicates: a,b and c. Cumulative N_2_O and CO_2_ fluxes given in Table 4, were calculated based on gas samples from ^15^N fertilizer treatments a,b and c. Fluxes of N_2_ and N_2_O_d_, as well as denitrification losses (N_2_ + N_2_O_d_), were calculated based on the gas samples from treatment c. Calculating cumulative N_2_O fluxes based on ^15^N fertilizer treatments a, b and c or c alone did not result in significant differences. The reduction of N_2_O by DMPP in the sandy CL was significant regardless of the calculation chosen.

The flux rates of N_2_O and CO_2_ were calculated from the slope of the linear increase in gas concentration during the closure period and were corrected for temperature and air pressure^20^. The ^15^N enrichment of the NO_3_^−^ pool undergoing denitrification (a_*p*_) and the fraction of N_2_ and N_2_O emitted from this pool (f_*p*_) were calculated following the equations given by Spott, *et al*.^43^ detailed in the supplementary material. The headspace concentrations of N_2_O and N_2_ were multiplied by the respective f_*p*_ values giving N_2_ and N_2_O produced via denitrification (referred to as N_2_ and N_2_O_d_), with their respective fluxes expressed in g N_2_ or N_2_O_d_ –N emitted g^−1^ soil day^−1^. Potential hybrid formation of N_2_ and N_2_O was found to be irrelevant^30^. The precision of the IRMS for N_2_ based on the standard deviation of atmospheric air samples (n = 18) at 95% confidence interval was 4.4 × 10^−7^ and 6.0 × 10^−7^ for ^29^R and ^30^R, respectively. The corresponding method detection limit ranged from 0.005 µg N_2_-N g^−1^ soil with a_*p*_ assumed at 50 atom % to 0.014 µg N_2_-N g^−1^ soil with a_*p*_ assumed at 20 atom %.

### Gross N transformations

Gross N transformations were quantified using a ^15^N tracing model^44^ (Fig. 1), which uses a Markov Chain Monte Carlo method optimizing the kinetic parameters for the various N transformations by minimizing the misfit between modeled and observed NH_4_^+^ and NO_3_^−^ concentrations and their respective ^15^N enrichments (soil microcosms a and b). The model considers five N pools including the NH_4_^+^ and NO_3_^−^ pool, a labile (N_lab_) and a recalcitrant (N_rec_) organic N pool, and a pool for NH_4_^+^ adsorbed to cation exchange sites (NH_4_^+^_ads_). These pools are defined by 10 simultaneous occurring gross N transformations calculated by zero-, first-order or Michaelis-Menten kinetics (Table 2): The mineralization of N_lab_ and N_rec_ to NH_4_^+^ (*M*_*nlab*_*, M*_*Nrec*_), the immobilization of NH_4_^+^ to N_lab_ and N_rec_ (*I*_*NH4-Nrec*,_ *I*_*NH4-Nlab*_), the adsorption (*A*_*NH4*_) and release *(R*_*NH4a*_) of NH_4_^+^ from NH_4_^+^_ads_, the oxidation of NH_4_^+^ to NO_3_^−^ (*O*_*NH4*_), referred to as autotrophic nitrification; the oxidation of *N*_*rec*_ to NO_3_^−^ (*O*_*Nrec*_), referred to as heterotrophic nitrification; dissimilatory NO_3_^−^ reduction to NH_4_^+^ (*D*_*NO3*_) and *I*_*NO3*_, the immobilisation of NO_3_^−^ to *N*_*rec*_. Total mineralization was calculated as the sum of *M*_*nlab*_ and *M*_*Nrec*_, total nitrification as the sum of *O*_*Nrec*_ and *O*_*NH4*_ and total immobilization of NH_4_^+^ as the sum of *I*_*NH4-Nrec*_ and *I*_*NH4-Nlab*_.

### Calculations and statistical analysis

The optimization routine used for the ^15^N tracing model gives a probability density function for each model parameter, which is used to calculated average values and standard errors of the mean. Average gross N transformation rates are obtained by integrating these values over the incubation period. Differences between N-transformations were assessed testing whether the 95% confidence intervals overlap^45^. The Benjamini Horchberg (BH) adjustment^46^ was performed to assess the effect of the different fertilization strategies on microbial C and N, DOC and *nosZ gene* abundance for each soil type. Analysis of variance was performed to assess differences in cumulative emissions of N_2_, N_2_O, total denitrification (N_2_ + N_2_O) and CO_2_ between soils within treatments and within soils between fertilization strategies. All values unless otherwise stated are given as mean ± standard error of the mean.

## Supplementary information

Supplementary information Effect of the nitrification inhibitor DMPP on N-turnover, the N<sub>2</sub>O reductase-gene <i>nosZ</i> and N<sub>2</sub>O: N<sub>2</sub> partitioning from agricultural soil.

## Data Availability

All data generated or analyzed during this study are included in this published article (and its Supplementary Information files).

## References

[1] Eric AD, David K (2014). Inventories and scenarios of nitrous oxide emissions. Environmental Research Letters.

[2] Akiyama H, Yan X, Yagi K (2010). Evaluation of effectiveness of enhanced‐efficiency fertilizers as mitigation options for N_2_O and NO emissions from agricultural soils: meta-analysis. Global Change Biology.

[3] Koci J, Nelson PN (2016). Tropical dairy pasture yield and nitrogen cycling: Effect of urea application rate and a nitrification inhibitor (DMPP). *Crop and Pasture*. Science.

[4] Menéndez S, Merino P, Pinto M, González-Murua C, Estavillo JM (2006). 3,4-Dimethylpyrazol phosphate effect on nitrous oxide, nitric oxide, ammonia, and carbon dioxide emissions from grasslands. Journal of environmental quality.

[5] Dougherty WJ, Collins D, Van Zwieten L, Rowlings DW (2016). Nitrification (DMPP) and urease (NBPT) inhibitors had no effect on pasture yield, nitrous oxide emissions, or nitrate leaching under irrigation in a hot-dry climate. Soil Research.

[6] Ruser R, Schulz R (2015). The effect of nitrification inhibitors on the nitrous oxide (N_2_O) release from agricultural soils-a review. Journal of Plant Nutrition and Soil Science.

[7] Baggs EM (2011). Soil microbial sources of nitrous oxide: recent advances in knowledge, emerging challenges and future direction. Current Opinion in Environmental Sustainability.

[8] Müller C, Laughlin RJ, Spott O, Rütting T (2014). Quantification of N2O emission pathways via a ^15^N tracing model. Soil Biology and Biochemistry.

[9] Butterbach-Bahl, K., Baggs, E. M., Dannenmann, M., Kiese, R. & Zechmeister-Boltenstern, S. Nitrous oxide emissions from soils: how well do we understand the processes and their controls? *Philosophical Transactions of the Royal Society B: Biological Sciences* **368** (2013). 10.1098/rstb.2013.0122 PMC3682742 23713120

[10] Friedl J (2018). Dissimilatory nitrate reduction to ammonium (DNRA), not denitrification dominates nitrate reduction in subtropical pasture soils upon rewetting. Soil Biology and Biochemistry.

[11] Friedl J, Scheer C, Rowlings DW, Mumford MT, Grace PR (2017). The nitrification inhibitor DMPP (3,4-dimethylpyrazole phosphate) reduces N_2_ emissions from intensively managed pastures in subtropical Australia. *Soil Biology &*. Biochemistry.

[12] Friedl J (2016). Denitrification losses from an intensively managed sub-tropical pasture – Impact of soil moisture on the partitioning of N_2_ and N_2_O emissions. Soil Biology & Biochemistry.

[13] Subbarao GV (2006). Scope and Strategies for Regulation of Nitrification in Agricultural Systems—Challenges and Opportunities. Critical Reviews in Plant Sciences.

[14] Ernfors M (2014). The nitrification inhibitor dicyandiamide increases mineralization-immobilization turnover in slurry-amended grassland soil. Journal of Agricultural Science.

[15] Shi X (2016). Effects of the Nitrification Inhibitor 3, 4-Dimethylpyrazole Phosphate on Nitrification and Nitrifiers in Two Contrasting Agricultural Soils. Applied and Environmental Microbiology.

[16] Dendooven L, Anderson JM (1995). Use of a “least square” optimization procedure to estimate enzyme characteristics and substrate affinities in the denitrification reactions in soil. Soil Biology & Biochemistry.

[17] Scheer C, Wassmann R, Kienzler K, Ibragimov N, Eschanov R (2008). Nitrous oxide emissions from fertilized, irrigated cotton (Gossypium hirsutum L.) in the Aral Sea Basin, Uzbekistan: Influence of nitrogen applications and irrigation practices. *Soil Biology &*. Biochemistry.

[18] Scheer C (2016). Effect of enhanced efficiency fertilisers on nitrous oxide emissions in a sub-tropical cereal cropping system. Soil Research.

[19] Rowlings DW, Scheer C, Liu S, Grace PR (2016). Annual nitrogen dynamics and urea fertilizer recoveries from a dairy pasture using ^15^N; effect of nitrification inhibitor DMPP and reduced application rates. *Agriculture, Ecosystems &*. Environment.

[20] Scheer C (2014). Impact of nitrification inhibitor (DMPP) on soil nitrous oxide emissions from an intensive broccoli production system in sub-tropical Australia. Soil Biology and Biochemistry.

[21] De Rosa D (2018). N_2_O and CO_2_ emissions following repeated application of organic and mineral N fertiliser from a vegetable crop rotation. Science of The Total Environment.

[22] Hallin S, Philippot L, Löffler FE, Sanford RA, Jones CM (2018). Genomics and Ecology of Novel N_2_O-Reducing Microorganisms. Trends in Microbiology.

[23] Giles M, Morley N, Baggs EM, Daniell TJ (2012). Soil nitrate reducing processes - drivers, mechanisms for spatial variation, and significance for nitrous oxide production. Frontiers in microbiology.

[24] Huang Y, Li Y, Yao H (2014). Nitrate enhances N_2_O emission more than ammonium in a highly acidic soil. Journal of Soils and Sediments.

[25] Torralbo F (2017). Dimethyl pyrazol-based nitrification inhibitors effect on nitrifying and denitrifying bacteria to mitigate N_2_O emission. Scientific reports.

[26] Wu D (2017). The effect of nitrification inhibitor on N_2_O, NO and N_2_ emissions under different soil moisture levels in a permanent grassland soil. Soil Biology and Biochemistry.

[27] Hatch D (2005). Laboratory study of the effects of two nitrification inhibitors on greenhouse gas emissions from a slurry-treated arable soil: impact of diurnal temperature cycle. Biology and Fertility of Soils.

[28] Marsden KA (2016). The mobility of nitrification inhibitors under simulated ruminant urine deposition and rainfall: a comparison between DCD and DMPP. Biology and Fertility of Soils.

[29] Keiblinger KM, Zehetner F, Mentler A, Zechmeister-Boltenstern S (2018). Biochar application increases sorption of nitrification inhibitor 3,4-dimethylpyrazole phosphate in soil. Environmental Science and Pollution Research.

[30] Gioacchini P (2002). Influence of urease and nitrification inhibitors on N losses from soils fertilized with urea. Biology and Fertility of Soils.

[31] Liu C, Wang K, Zheng X (2013). Effects of nitrification inhibitors (DCD and DMPP) on nitrous oxide emission, crop yield and nitrogen uptake in a wheat–maize cropping system. Biogeosciences.

[32] Azam F, Müller C, Weiske A, Benckiser G, Ottow J (2002). Nitrification and denitrification as sources of atmospheric nitrous oxide – role of oxidizable carbon and applied nitrogen. Biology and Fertility of Soils.

[33] Harty MA (2017). Gross nitrogen transformations in grassland soil react differently to urea stabilisers under laboratory and field conditions. Soil Biology and Biochemistry.

[34] Scheer C, Meier R, Brüggemann N, Grace PR, Dannenmann M (2016). An improved ^15^N tracer approach to study denitrification and nitrogen turnover in soil incubations. Rapid Communications in Mass Spectrometry.

[35] Senbayram M, Chen R, Budai A, Bakken L, Dittert K (2012). N_2_O emission and the N_2_O/(N_2_O + N_2_) product ratio of denitrification as controlled by available carbon substrates and nitrate concentrations. *Agriculture, Ecosystems &*. Environment.

[36] Friedl, J., Scheer, C., Rowlings, D. W., Trappe, J. & Grace, P. Nitrogen turnover and N_2_: N_2_O partitioning from agricultural soils–a simplified incubation assay. *International Nitrogen Initiative Conference, “Solutions to improve nitrogen use efficiency for the world”*, Retrieved from http://www.ini2016.com/conference-proceedings-2012 (2016).

[37] Shand CA, Williams BL, Coutts G (2008). Determination of N-species in soil extracts using microplate techniques. Talanta.

[38] Hood-Nowotny R, Hinko-Najera Umana N, Inselbacher E, Oswald-Lachouani P, Wanek W (2010). Alternative Methods for Measuring Inorganic, Organic, and Total Dissolved Nitrogen in Soil. Soil Science Society of America Journal.

[39] Stark JM, Hart SC (1996). Diffusion technique for preparing salt solutions, Kjeldahl digests, and persulfate digests for nitrogen-15 analysis. Soil Science Society of America Journal.

[40] Henry S, Bru D, Stres B, Hallet S, Philippot L (2006). Quantitative Detection of the nosZ Gene, Encoding Nitrous Oxide Reductase, and Comparison of the Abundances of 16S rRNA, narG, nirK, and nosZ Genes in Soils. Applied and Environmental Microbiology.

[41] Vance E.D., Brookes P.C., Jenkinson D.S. (1987). An extraction method for measuring soil microbial biomass C. Soil Biology and Biochemistry.

[42] Brookes, P., Kragt, J., Powlson, D., Jenkinson, D. J. S. B. &Biochemistry. Chloroform fumigation and the release of soil nitrogen: the effects of fumigation time and temperature. **17**, 831–835 (1985).

[43] Spott, O., Russow, R., Apelt, B. & Stange, C. F. A ^15^N-aided artificial atmosphere gas flow technique for online determination of soil N_2_ release using the zeolite Köstrolith SX6®. *Rapid Communications in Mass Spectrometry: An International Journal Devoted to the Rapid Dissemination of Up‐to‐the‐Minute Research in Mass Spectrometry* **20**, 3267–3274 (2006). 10.1002/rcm.2722 17044127

[44] Müller C, Rütting T, Kattge J, Laughlin RJ, Stevens RJ (2007). Estimation of parameters in complex ^15^N tracing models by Monte Carlo sampling. Soil Biology & Biochemistry.

[45] Rütting T, Clough TJ, Müller C, Lieffering M, Newton PC (2010). Ten years of elevated atmospheric carbon dioxide alters soil nitrogen transformations in a sheep‐grazed pasture. Global Change Biology.

[46] Benjamini Y, Hochberg Y (1995). Controlling the False Discovery Rate: A Practical and Powerful Approach to Multiple Testing. Journal of the Royal Statistical Society. Series B (Methodological).

