# Supplementary material for: Effect of the nitrification inhibitor 3,4-dimethylpyrazole phosphate (DMPP) on N-turnover, the N2O reductase-gene nosZ and N2O:N2 partitioning from agricultural soils
Source: Sci Rep. 2020 Feb 12;10:2399. doi: 10.1038/s41598-020-59249-z (PMC7016175; doi:10.1038/s41598-020-59249-z)
Supplement: Supplementary file 1 — Supplementary information Effect of the nitrification inhibitor DMPP on N-turnover, the N2O reductase-gene nosZ and N2O: N2 partitioning from agricultural soil. [file 41598_2020_59249_MOESM1_ESM.pdf]

Effect of the nitrification inhibitor 3,4-dimethylpyrazole phosphate (DMPP) on N-turnover, the N_2O reductase-gene *nosZ* and $N_2O:N_2$ partitioning from agricultural soils

Supplementary Material

Johannes Friedl[1,*] [1], Clemens Scheer[1], David W. Rowlings [1], Evi Deltedesco[2], Markus Gorfer[3], Daniele De Rosa[1], Peter R. Grace[1], Christoph Müller[4,5] and Katharina M. Keiblinger[2]

[1] Institute for Future Environments, Queensland University of Technology, Brisbane, QLD 4000, Australia
[2] University of Natural Resources and Life Sciences Vienna, Department of Forest and Soil Sciences, Institute of Soil Research, Vienna, Austria
[3] AIT Austrian Institute of Technology, Center for Health & Bioresources, Tulln, Austria
[4]Department of Plant Ecology (IFZ), Justus-Liebig University Giessen, Germany
[5] School of Biology and Environmental Science, University College Dublin, Belfield, Dublin, Ireland

[*]corresponding author: johannes.friedl@qut.edu.au

1.1 N_2 and N_2O flux calculations

Assuming that N_2 and N_2O originate from the same NO_3^- pool undergoing denitrification, N_2 fluxes are calculated based on the increase of $^{15}N_2$ in the chamber headspace and the ^{15}N enrichment of the NO_3^- pool, derived from ^{15}N-N_2O [1,2]. For N_2 and converted N_2O, the ion currents at *m/z* 28, 29 and 30 enabled the molecular ratios ^{29}R ($^{29}I/^{28}I$) and ^{30}R ($^{30}I/^{28}I$) to be calculated. The ion currents (I) at *m/z* 44, 45, and 46 enabled the molecular ratios ^{45}R ($^{45}I/^{44}I$) and ^{46}R ($^{46}I/^{44}I$) to be calculated for N_2O. ^{45}R and ^{46}R were then corrected for oxygen using the following equations given by Bergsma et al. (2001)[3]:

$$^{29}R = {}^{45}R - {}^{17}R \tag{1}$$

$$^{30}R = {}^{46}R - {}^{29}R * {}^{17}R - {}^{18}R \tag{2}$$

where ^{17}R =0.000373 and ^{18}R is 0.00200523

The ^{15}N enrichment of the NO_3^- pool undergoing denitrification (a_p) and the fraction of N_2 and N_2O emitted from this pool (f_p) were calculated following the equations given by Spott et al. (2006)[4]

$$f_p = \frac{a_m - a_{bgd}}{a_p - a_{bgd}} \tag{3}$$

where a_{bgd} is the ^{15}N abundance of the atmospheric background and a_m is the measured ^{15}N abundance of N_2 and N_2O,

$$a_m = \frac{^{29}R + 2 * {}^{30}R}{2 * (1 + {}^{29}R + {}^{30}R)} \tag{4}$$

a_p is the ^{15}N abundance of the NO_3^- pool undergoing denitrification,

$$a_p = \frac{^{30}x_m - a_{bgd} - a_m}{a_m - a_{bgd}} \tag{5}$$

and $^{30}x_m$ is the measured fraction of m/z 30 in N_2:

$$^{30}x_m = \frac{^{29}R + 2 * {}^{30}R}{2 * (1 + {}^{29}R + {}^{30}R)} \tag{4}$$

Comparing the NO_3^- pool undergoing denitrification calculated from the isotopologues of N_2O vs. N_2 showed no significant differences for a_p, confirming the assumption that both N_2 and N_2O_d were emitted from the same NO_3^- of the enrichment a_p. Following Stevens and Laughlin (2001) [2], a_p derived from converted N_2O was then used to calculate N_2 fluxes. Both N_2 and N_2O derived from denitrification were calculated by multiplying the respective headspace concentration with the respective value of f_p and expressed in g N_2 or N_2O_d –N emitted g^{-1} soil day^{-1}. Potential hybrid formation of N_2 and N_2O was investigated using the equations detailed in Spott et al. (2006)[30] but was found to be irrelevant.

1.2 Standards for *nosZ* quantitative polymerase chain reaction (qPCR) analyses

Standards for qPCR were prepared by amplification of reference *nosZ* fragments from an agricultural soil with the same primers as used for qPCR[5]. Amplicons were cloned into pJet1.2 Blunt Cloning Vector (ThermoFisher Scientific) according to manufacturer's instructions. Inserts were amplified and sequenced with pJet1.2 forward and reverse sequencing primers to confirm amplification of partial *nosZ* genes. From appropriate clones, PCR products with pJet1.2 forward and reverse sequencing primers were amplified, purified with the Qiaquick PCR Purification Kit (Qiagen, Venlo, Netherlands) and quantified with iQuant Broad Range dsDNA Quantitation Kit (GeneCopoeia, MD, USA). Gene copy numbers of the standard were calculated from concentration and length of PCR products.

References:

1 Mulvaney, R. Determination of ^{15}N-labeled dinitrogen and nitrous oxide with triple-collector mass spectrometers. *Soil Science Society of America Journal* **48**, 690-692 (1984).

2 Stevens, R. J. & Laughlin, R. J. Lowering the detection limit for dinitrogen using the enrichment of nitrous oxide. *Soil Biol. Biochem.* **33**, 1287-1289, doi:http://dx.doi.org/10.1016/S0038-0717(01)00036-0 (2001).

3 Bergsma, T. T., Ostrom, N. E., Emmons, M. & Robertson, G. P. Measuring simultaneous fluxes from soil of N2O and N2 in the field using the 15N-gas "nonequilibrium" technique. *Environmental Science and Technology* **35**, 4307-4312, doi:10.1021/es010885u (2001).

4 Spott, O., Russow, R., Apelt, B. & Stange, C. F. A 15N-aided artificial atmosphere gas flow technique for online determination of soil N2 release using the zeolite Köstrolith SX6®. *Rapid Communications in Mass Spectrometry: An International Journal Devoted to the Rapid Dissemination of Up-to-the-Minute Research in Mass Spectrometry* **20**, 3267-3274 (2006).

5 Henry, S. *et al.* Quantitative detection of the nosZ gene, encoding nitrous oxide reductase, and comparison of the abundances of 16S rRNA, narG, nirK, and nosZ genes in soils. **72**, 5181-5189 (2006).
